# Modeling COVID-19 Impacts and Response Strategies in the Construction Industry: PLS–SEM Approach

**DOI:** 10.3390/ijerph19095326

**Published:** 2022-04-27

**Authors:** Afiqah R. Radzi, Rahimi A. Rahman, Saud Almutairi

**Affiliations:** 1 Faculty of Built Environment, University of Malaya, Kuala Lumpur 50603, Malaysia; s2133585@siswa.um.edu.my; 2 Faculty of Civil Engineering Technology, Universiti Malaysia Pahang, Gambang 26300, Malaysia; 3 General Educational Development, Daffodil International University, Dhaka 1341, Bangladesh; 4 Unaizah College of Engineering, Qassim University, Buraydah 51431, Saudi Arabia

**Keywords:** construction industry, COVID-19, pandemic impact, pandemic response, strategic planning

## Abstract

Policymakers are developing response strategies to reduce the impacts of COVID-19. However, developing response strategies without considering their relationships with the impacts of COVID-19 is ineffective. This study aims to model the causal relationships between COVID-19 impacts and response strategies in the construction industry, using Malaysia as a case study. To achieve this, a systematic literature review and semi-structured interviews with forty industry professionals were conducted, yielding 12 impacts and 22 response strategies. The impacts and strategies were inserted into a survey, and 107 valid responses were received. Exploratory factor analysis (EFA) was conducted to group the impacts and strategies. Then, partial least-squares structural equation modeling (PLS–SEM) was employed to identify the causal relationship between the impacts and strategies. The EFA results indicate that the underlying impacts are project- or material-related, and the underlying strategies are market stability and financial aid, supply chain and project support, and information and legislation. The PLS–SEM results indicate that supply chain and project support are required to address material-related impacts, and market stability and financial aid are required to address project-related impacts. This is the first paper that models the relationships between COVID-19 impacts and response strategies in the construction industry.

## 1. Introduction

The construction industry is a large sector, accounting for 13% of the global gross domestic product (GDP) [1]. Thus, any reduction in construction sector performance negatively affects the national economy [2]. The coronavirus, known as COVID-19, has affected the world economy. Apart from the widespread health crisis, COVID-19 has negatively impacted the construction industry [3,4]. Construction organizations with high debt and low cash reserves face liquidity as a result of the pandemic [3]. The pandemic has affected construction activities by disrupting the global supply chain and creating material and labor shortages [4]. Current, planned, and new construction projects face delays, suspensions, and cancelations [5,6]. Moreover, response strategies undertaken by policymakers to slow the spread of COVID-19 or flatten the curve, such as social distancing and quarantines, have created uncertainties and challenges for construction projects and at job sites [7]. The construction industry is one of the industries most affected by the pandemic, with this resulting in significant effects on the economy [8]. Finding approaches to reduce the impacts of COVID-19 is crucial for preventing negative economic growth and economic recession.

Policymakers are developing comprehensive response strategies to address COVID-19. Financial assistance is provided to small- and medium-sized businesses through investment loan packages [9]. In Australia, an emergency supply registration portal was created for suppliers who could deliver critical supplies, raw materials, or manufacturing capabilities during the pandemic [10]. Due to a lack of understanding, these response strategies have been established for all industries rather than for each industry. The construction industry has unique characteristics, including a complicated nature of operations, tight schedules with limited budgets, and many types of workers and organizations [11], resulting in different COVID-19 impacts and response strategies. Specific response strategies must be developed to address the impacts of COVID-19 on the construction industry. The response strategies in recovery and resilience plans will only be justifiable, effective, and efficient by fully considering the underlying industry-specific characteristics.

Researchers are investigating COVID-19 impacts on the construction industry [12,13,14] as well as response strategies to address them [15,16]. Moreover, some works have modeled the relationship between COVID-19 safety protocols and projects’ economic performance [17], as well as the relationship among social capital, knowledge creation, and construction productivity during the COVID-19 era [18]. However, from the mentioned studies, it is evident that prior works have not considered examining the relationship between COVID-19 impacts and response strategies. Understanding the relationship between the impacts and response strategies can assist industry stakeholders and policymakers in developing appropriate and suitable response strategies. Therefore, there is a need to investigate the relationship between COVID-19 impacts and response strategies.

This study aims to bridge this knowledge gap by modeling the causal relationships between COVID-19 impacts and response strategies in the construction industry, using Malaysia as a case study. The objectives of this study include identifying (1) the underlying impacts of COVID-19 on the construction industry, (2) the underlying government response strategies to COVID-19 for the construction industry, and (3) the relationships between these impacts and response strategies. This study deepens the understanding of the relationships between the negative impacts of COVID-19 and effective response strategies to address them, helping policymakers and industry stakeholders to identify the best response strategies to COVID-19 and to avoid recurring impacts in future pandemics. Sustaining construction during hardships can avoid repercussions in local economies that can result in economic recessions or collapses.

## 2. Background

### 2.1. COVID-19 Impacts

COVID-19 has caused various issues and problems for the modern world’s healthcare, economic, and social systems. The construction industry, as well as other industries, have been negatively impacted by the pandemic. COVID-19 not only affects the construction industry in terms of construction projects, but also the workforce and construction organizations. For example, ref [12] conducted questionnaire surveys and revealed that the impacts of COVID-19 include the suspension of projects, time overrun, cost overrun, and financial impact. Additionally, [13] investigated the early impacts of COVID-19 on the US construction industry, including project delays. Ref [19] identified the impacts of COVID-19 on tunnel construction projects, i.e. schedule delays and cost overruns. The study in ref [14] conducted a questionnaire survey in Kuwait and revealed that COVID-19 impacts construction projects by shortening the daily working period. The authors of ref [20] identified COVID-19 impacts on building construction projects; the impacts include project timeline, labor, logistics, late payments, increased cost, and reduced projects. The study conducted in ref [21] investigates the COVID-19 impacts on infrastructure construction projects: cost, income, process, and management.

COVID-19 not only affects construction projects, but also causes negative impacts on the construction workers in the field as well as in the office. The author in ref [22] conducted a questionnaire to identify the impact of COVID-19 on civil engineers in Jordan. The result shows that some of the engineers fear that they might lose their job due to lockdown. The authors of ref [23] conducted a systematic review to identify the impacts of COVID-19 on the field and office workers in the construction industry. The study categorizes the impacts into organizational, economic, psychological, individual, and moderating factors. Furthermore, ref [24] conducted face-to-face interviews to investigate the experience of construction workers during COVID-19. The results show that construction workers experience unexpected work suspension and have suffered psychologically and emotionally from homesickness due to the travel ban. Construction organizations also experienced negative impacts due to COVID-19. For example, ref [19] revealed that site accessibility, worker availability, material shortage, and panic of surrounding residents had significantly hindered construction progress. In addition, ref [13] identify some pandemic impacts affecting construction organizations, such as the inability to secure materials on time, a reduction in productivity rates, and material price escalations. In addition to that, the impacts that affect construction organization in Ghana include decreased work rate, delays in payments, and increased material cost due to border closure [5].

### 2.2. Pandemic Response

Policymakers are developing response strategies to address the pandemic’s impacts. For example, policymakers in Sri Lanka [9] and Australia [10] have developed response strategies to overcome the pandemic’s impacts. At the organizational level, the response strategies include keeping standard operating procedures, establishing successful relationships with suppliers, and working in shifts [15]. Furthermore, ref [13] suggested some response strategies, such as creating teams to review the pandemic and suggesting recommendations, as well as capitalizing on available government relief programs. The study conducted in ref [25] reviewed related articles and proposed response strategies for the future of the construction industry post-COVID-19. There are eleven strategies that can be used to develop pandemic resiliency among construction organizations; these strategies include portfolio diversification, collaborative contracting methods, industrialized construction, circular economy, remote working, integrated design management using building information modeling (BIM), staffing and skills training, reversible building design, augmented reality, automation, three-dimensional printing, and lean construction.

Ref [26] discovered that policymakers should declare the pandemic a force majeure event, as COVID-19 poses a serious risk to the AEC industry. The study in ref [16] concluded that building construction projects demand financial aid and information to combat the pandemic’s impact. Some prior works have also come out with response strategies to overcome the pandemic’s impacts. Ref [23] discovered that providing sanitizers and washing stations at construction sites, putting up signs to redefine worksite safety, ensuring safe distances between workers, and using effective technologies can improve worker safety and project productivity. According to ref [27], separating sick workers, performing daily checks for COVID-19 signs, prohibiting hugs and handshakes, displaying health advice posters and infographics, and supplying face masks to workers are all effective response strategies in decreasing transmission risks. The authors in ref [28] identified three effective techniques, including screening, site access, and the on-site management of material and delivery of equipment.

### 2.3. COVID-19 in the Malaysian Construction Industry

COVID-19 has emerged in society, devastatingly impacting many industries, including the construction industry. The construction industry contributes significantly to Malaysia’s economic growth, with an annual total return of MYR204 billion on construction projects [29]. Like many other nations, the Malaysian government enforced movement restrictions during the outbreak due to the constant and rapid growth of COVID-19 cases. As a result, construction production declined by 13.1 percent yearly to MYR31.4 billion in the third quarter of 2020 [30]. Material supply shortages also occurred in many areas due to travel restrictions or post-travel quarantines [31]. Another serious issue is human resources, as many foreign workers cannot reenter Malaysia or leave their home countries due to travel restrictions. Construction organizations and contractors were forced to deal with labor scarcity resulting from restrictions on the admittance of foreign workers. In addition, contractors had to cope with contract delays or increased expenses, as well as other changes that needed to be made. Material shortages are frequently caused by changes and delays in material acquisition, site operations, and supply chain issues [20].

Researchers in Malaysia are conducting research to investigate and mitigate the pandemic’s impact on the Malaysian construction industry. The research in ref [21] identified five critical pandemic impacts, including reduced construction productivity, reduced foreign investment in the construction industry, reduced demand for construction-related works, disruption in the supply chain, and a reduced number of public projects. Ref [32] investigated COVID-19’s challenges to workforce productivity and strategies to overcome the pandemic’s impact. The most significant problem faced by workers is challenges in adopting new norms on site, followed by workforce shortage, planning and schedule disruption, workforce health and workforce management issues. While the most effective strategy is increasing the use of communication technology, followed by redefining risk and safety management on-site and adjusting working spaces based on the standard operating procedure. The consequences of the lockdown implemented by the Malaysian government (i.e., the Movement Control Order or MCO) towards construction project success were explored in ref [33]. The result shows that MCO has negatively impacted project success in terms of regulation compliance, safety, additional time for project delivery, increase in development cost, limited human resources supplies, and limited resource availability on-site. The work in ref [16] identified government-level response strategies using questionnaire surveys. The study concluded that there are four critical response strategies for small–medium enterprises, including forming a special task force to provide support in maneuvering COVID-19, providing infrastructure investment budgets to local governments, developing employee assistance programs that fit all types of working groups, and diversifying existing supply chain. Large enterprises have two distinct critical response strategies, including providing help in digitizing existing construction projects and mandating COVID-19 as force majeure.

### 2.4. Knowledge Gap

This subsection synthesizes the knowledge gaps that exist in the current literature to support the rationale for conducting the study. Although prior works have studied COVID-19’s impacts and response strategies in the construction industry, much remains unknown, as COVID-19 is a relatively new topic. In addition, previous works lack insights into the relationships between COVID-19 impacts and response strategies. Prior works only focused on identifying the impacts and response strategies independently, without connecting them. Developing appropriate response strategies is critical for addressing the targeted impacts. The current study leverages the knowledge gap by identifying (1) the underlying impacts of COVID-19 on the Malaysian construction industry, (2) the construction industry response strategies to COVID-19, and (3) the relationships between the underlying impacts and response strategies.

## 3. Methodology

### 3.1. Survey Development

**Figure 1:**
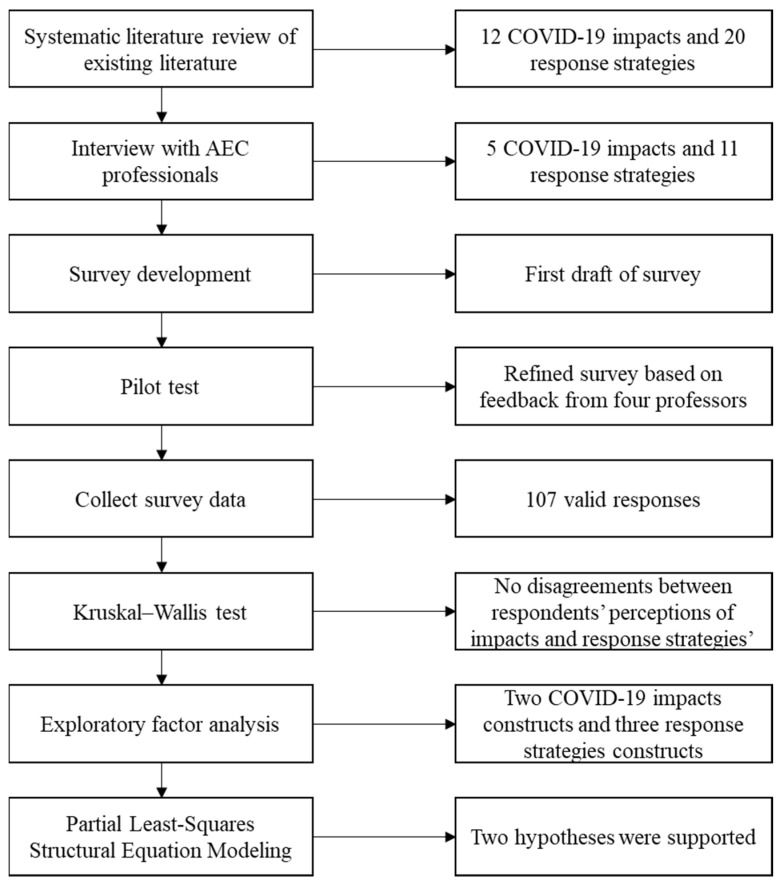
Study framework.

Questionnaire surveys systematically collect quantitative data using random samples [34]. This approach has been frequently used to obtain expert opinions in the field of construction management [35,36]. Figure 1 shows the broad framework of this study.

#### 3.1.1. Systematic Literature Review

This study conducted a systematic literature review (SLR) using the Preferred Reporting Items for Systematic Review and Meta-Analyses (PRISMA) protocol guidelines to generate a list of potential COVID-19 impacts and response strategies. The first search was conducted using the ‘title/abstract/keyword’ feature in the Scopus database using the terms ‘COVID’ and ‘construction industry’ OR ‘construction industries’ OR ‘construction management’ OR ‘project management’ OR ‘construction engineering’ OR ‘construction project’ OR ‘construction projects.’ This study also looked for papers related to other industries to identify additional COVID-19 impacts and response strategies. Then, a second search was conducted using the keywords ‘COVID’ and ‘impact’ or ‘response.’ This search limited papers to the subject areas of ‘business, management, and accounting’ and ‘economics, econometrics, and finance’ to narrow the scope of the business and economics body of knowledge. Based on the search code, 519 articles were retrieved. There were no duplicates between both searches. All selected articles were peer-reviewed publications from well-recognized journals. Conference papers and thesis dissertations were not included due to their quality. Furthermore, not all articles were related to COVID-19 impacts and response strategies. The unrelated articles were excluded after examining their abstracts and full contents. In the end, 72 articles were found and analyzed (see Appendix A).

#### 3.1.2. Interview

In addition to the SLR, forty semi-structured interviews were conducted by phone with AEC professionals to collect COVID-19 impacts and response strategies. Interview forms were used while interviewing the industry professionals. The interviews were conducted to identify additional COVID-19 impacts and response strategies (i.e., variables) missing from the current body of knowledge [20]. To ensure the reliability of the interview results, the interviews were conducted with industry professionals in senior or managerial positions with at least five years of experience in the construction industry. The interviews started with an introduction that explained the purpose of the interview and the topic of the discussion. Then, the interview questions were asked: (1) What problems the construction industry is facing post-COVID-19? (2) What strategies are effective in solving those problems? and (3) What government assistances are effective in solving those problems? The questions were followed by additional questions depending on the interviewees’ responses. The follow-up questions were designed to obtain a deeper understanding of the information that they gave and to ensure their statements were understood correctly [20]. If the participant could not respond or elaborate on the questions asked, the interviewer tried to rephrase the interview question in another way and gave time for a response. The interviewer encouraged the interviewees to continue if they had started on an answer without finishing their explanation. The interviewer ended the interviews by thanking the interviewees. After each interview, a summary was generated and sent to the respondent for validation. Then, the interview data was analyzed to generate a list of COVID-19 impacts and response strategies using the thematic analysis technique as described in [37].

#### 3.1.3. Survey Design

Using the data collected from the SLR and the interviews, the survey was developed. Impacts and response strategies with similar meanings were combined, resulting in 12 impacts and 22 response strategies. The study objectives and contact details were displayed on the front page of the survey; the survey followed in two parts. The first part included questions about the backgrounds and organizations of the respondents, which was essential for assessing their reliability. The second part consisted of the twelve identified COVID-19 impacts. Respondents were asked to rank the importance of the COVID-19 impacts on the construction industry on a five-point Likert scale (1 = not critical, 2 = less critical, 3 = neutral, 4 = critical, 5 = extremely critical). This scale was adopted owing to its short length and effectiveness in evaluating variables through questionnaire surveys [38,39]. The third part included the twenty-two identified response strategies. Respondents were asked to score the effectiveness of the response strategies on a five-point Likert scale (1 = very low, 2 = low, 3 = neutral, 4 = high, and 5 = very high). The five-point Likert scale is popular for its ability to provide clear information [38,39,40]. At the end of the survey, respondents were given space to describe and assess any additional COVID-19 impacts and response strategies. Appendix B shows the final form of the survey.

#### 3.1.4. Pilot Test

A pilot test can detect any issues in the design and instrumentation of a survey [41]. Furthermore, the feedback received from the pilot test is crucial in improving the quality and determining the time required to finish the survey [42]. Therefore, a pilot test was conducted involving four professors with more than ten years of expertise in construction management to eliminate unclear statements and ensure the proper use of technical jargon. The pilot test participants were provided with the survey form and were asked to express their views about each of the items in the survey. They were also free to proceed to detailed modifications, including ‘add’, ‘delete’, or ‘combine’. By the fourth participant, the authors concluded that the information retrieved had reached data saturation. Data saturation occurs when a researcher may realistically assume further data collection would yield identical results and confirm emerging themes and conclusions [43]. Finally, the survey was finalized based on feedback from the pilot test.

### 3.2. Data Collection

The target population for the survey included all industry professionals with the required expertise and experience in the Malaysian construction industry. The nonprobability sampling method was used in this study, as the individuals within the target population could not be listed or specifically identified (i.e., no sampling frame) [38,39,44]. To reach the target population, the snowball sampling technique was used, as it enables data collection from industry experts via referrals and social networks [38,39,45]. To determine the initial respondents, AEC professionals directly involved in the construction industry were contacted. Respondents were asked to indicate others they deemed appropriate for the survey based on industrial or academic experience. Two follow-ups were sent to the target populations two weeks after the first contact to increase the survey success rate. As a result, a total of 107 valid responses were obtained.

**Figure 2:**
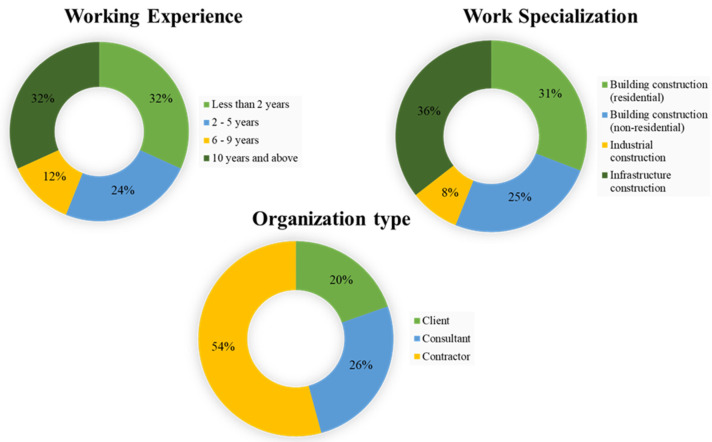
Respondent profile.

Figure 2 presents the respondents’ background information. Respondents were classified according to their years of experience, work specialization, and organizational type. All respondents were construction industry professionals, including project managers, engineers, architects, and quantity surveyors with adequate knowledge of the construction industry. The distribution of respondents with less than 2 years, 2–5 years, 6–9 years, and greater than 9 years of working experience was approximately 32%, 24%, 12%, and 32%, respectively. These results reflect great experience in construction; more than half of the respondents can be considered experts with at least two years of experience in construction projects. In addition, 36% of the respondents specialized in infrastructure construction, 31% in residential building construction, 25% in non-residential building construction, and 8% in industrial construction. Most respondents were contractors (54%), followed by consultants (26%) and clients (20%).

## 4. Analysis and Results

Statistic Package for the Social Sciences (SPSS) version 23.0 was used to conduct exploratory factor analysis, and SmartPLS 3 was used to statistically test the hypotheses based on structural equation modeling using the partial least squares (PLS) approach.

### 4.1. Common Method Variance

Common method variance (CMV) is a potential problem caused by the use of a single measurement method to measure constructs with a causal relationship. Cross-sectional research using self-reported questionnaire data is a concern. Harman’s single-factor test can measure CMV using the factor analysis technique [46]. In the test, constructs with a total variance of less than 50% indicate that CMV does not affect the data [44]. In this study, the greatest total variance for any single construct was 44.243%. Thus, CMV was not a factor, and no single construct dominated the results [46].

### 4.2. Kruskal-Wallis Test

As the collected data were not always normally distributed, nonparametric tests were used for analysis. The Kruskal–Wallis test was used to determine any significant differences in the perception regarding the impacts and response strategies among clients, contractors, and consultants. According to [47], a significant difference is indicated when the asymptotic significance value is less than 0.05. The Kruskal–Wallis test results showed asymptotic significance values greater than 0.05, indicating no significant differences among the respondents.

### 4.3. Exploratory Factor Analysis

Exploratory factor analysis (EFA) is a data-driven approach used to determine the construct structure and assess its internal reliability. EFA helps to regroup and reduce many interrelated variables into a smaller and more relevant set of constructs [48]. The study in [49] suggested that researchers use EFA when there are no prior hypotheses regarding factors or patterns of measured variables.

The ratio of the sample size to the number of variables was used to determine the sample size for the EFA method. The ratio of the sample size to the number of variables was 8.91 for pandemic impacts, which is greater than the recommended value of 5.00 [50]. The ratio for the pandemic response strategies was 4.86, which is slightly below 5.00. Thus, the sample size for this study was considered to be adequate.

The Kaiser–Meyer–Olkin (KMO) measure of sampling adequacy and Bartlett’s test of sphericity values were used to determine the appropriateness of the data for EFA. The KMO test determines if values are sufficiently distributed in the measurement sample of the factor analysis, for which a minimum KMO coefficient of 0.8 is required [49]. In this study, the calculated KMO values for the pandemic’s impacts and response strategies are 0.880 and 0.840, greater than the required KMO value of 0.50 [50]. On the other hand, a large Bartlett’s test sphericity and a small level of associated significance (*p* < 0.05) indicate that the population correlation matrix is not an identity matrix, and that EFA is appropriate [51]. The significance levels of the impacts and response strategies in Bartlett’s test were <0.001. These tests confirmed that the data were suitable for factor analysis.

The principal axis factoring (PAF) was used as an extraction method because it yields more stable loadings than other factor extraction methods for EFA [52]. This approach has been used in other works [39,53,54]. The factor-loading threshold value for identifying a construct is 0.50 [55]. Thus, indicators with factor loading less than 0.5 were removed. Nine of the twelve COVID-19 impacts were successfully loaded into the two underlying constructs with factor loadings greater than 0.50, with a total variance of 53.644%, as shown in Table 1.

Fourteen of the twenty-two response strategies were extracted from the three constructs with factor loadings greater than 0.50, with a total variance of 63.009%, as also shown in Table 1. According to [56], the construct label can be assigned based on variables with higher factor loadings or on the entire set of variables. The Cronbach’s α reliability test was run to ensure that the factors were appropriately grouped. Table 1 shows the Cronbach’s alpha coefficients, ranging from 0.646 to 0.856—greater than the required minimum of 0.60 [57], indicating that each construct exhibited good internal consistency.

### 4.4. Hypotheses for Structural Model

Based on the EFA, six hypotheses were developed to examine the relationships between the COVID-19 impacts and response strategies. The hypotheses can be separated into two groups, impacts related to projects (H1–H3) and materials (H4–H6), as shown below:

**Hypothesis** **1** **(H1).** *Project-related impacts influenced the need for market stability and financial aid.*

**Hypothesis** **2** **(H2).** *Project-related impacts influenced the need for supply chain and project support.*

**Hypothesis** **3** **(H3).** *Project-related impacts influenced the need for information and legislation.*

**Hypothesis** **4** **(H4).** *Material-related impacts influenced the need for market stability and financial aid.*

**Hypothesis** **5** **(H5).** *Material-related impacts influenced the need for supply chain and project support.*

**Hypothesis** **6** **(H6).** *Material-related impacts influenced the need for information and legislation.*

### 4.5. Partial Least-Squares Structural Equation Modeling

The hypotheses were tested using structural equation modelling (SEM). SEM can be used to measure observed variables directly, whereas latent variables can be inferred from observed variables. Measurement models and structural models make up a structural equation model. The relationship between each observed variable and its latent variable is illustrated in a measurement model. The relationships between latent variables are represented in a structural model. Covariance-based SEM (CB–SEM) and partial least-squares SEM (PLS–SEM) are the two forms of SEM. PLS–SEM was chosen over CB–SEM, because it is better able to handle non-normal datasets and small sample sizes [58]. It is also best used for exploratory research with theoretical models that are not well-developed [59].

PLS–SEM generates a set of measurement models and a structural model. First, the measurement model’s validity is evaluated using composite reliability, measurement item loadings on corresponding constructs, and average variance extracted (AVE). Internal consistency reliability is measured using composite reliability, which should be greater than 0.7 [60]. The recommended threshold for the loadings of the measurement items indicator is 0.70 [61]. The convergent validity is assessed using the AVE, which should have a value greater than 0.5 [60]. After that, discriminant validity is assessed. The degree to which a given construct differs from other constructs is known as discriminant validity [62]. For adequate discriminant validity, the square root of the AVE of each construct should be higher than the inter-construct correlation, and a measurement item’s loading on its respective construct should exceed the cross-loadings [61]. Finally, the structural model validity is assessed using collinearity issues (inner variance inflation factor values), the significance and relevance of the structural model relationships, the coefficient of determination (R2), and the effect sizes (f^2^).

According to the rule for determining the PLS–SEM minimum sample size, the sample size should be ten times the largest number of structural paths directed at a particular latent construct in the structural model [58]. Based on the measurement model, a minimum sample size of 20 was required for this study. Furthermore, a minimum sample size of 50 can be considered sufficient for PLS–SEM [63]. As the total number of responses (107) exceeds the both suggested thresholds (i.e., 20 and 50 respondents), the data can be considered suitable for PLS–SEM.

#### 4.5.1. Assessment of Measurement Model

Convergent validity and discriminant validity must be assessed when evaluating reflective measurement models in PLS–SEM. The structural model can be examined once the measurement model’s reliability and validity have been established.

#### 4.5.2. Convergent Validity

**Figure 3:**
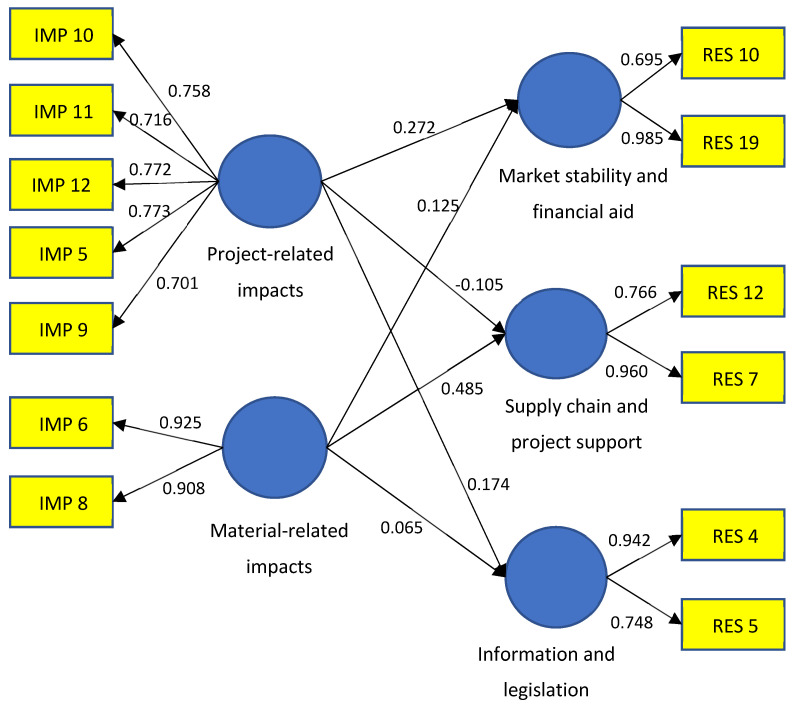
Measurement model.

Factor loadings, AVE, and composite reliability (CR) were used to assess the convergent validity of the reflective constructs, as described by [64]. The measurement model assessment is presented in Table 2 and Figure 3. Measurement indicators with factor loadings less than 0.7 were removed from the study. The process was repeated until a reliable and valid measurement model was produced. In Table 2, all observed variables had satisfactory loading except for RES10. RES10 was not removed, although it had loadings less than 0.70 to avoid any construct being measured with a single item [65]. The composite reliability values describe how well the construct indicators represent the latent construct and indicate internal consistency. All values were well above the cutoff value of 0.70 [64]. The AVE measures convergent reliability by computing the variance of its indicators; the recommended AVE value is ≥0.50 [66]. Cronbach’s alpha evaluates indicator reliability and should be greater than 0.6 [57]. The model provides sufficient evidence of convergent validity.

#### 4.5.3. Discriminant Validity (Fornell–Larcker Criterion)

After evaluating the convergent validity of the measurement model, the next step was to evaluate its vertical collinearity. This evaluation involves estimating the discriminant validity using the Fornell–Larcker criterion. A construct should share more variance with its measurements than it does with other constructs in the model. In Table 3, the highest correlation for a construct is the correlation with itself. The diagonal values in Table 3 represent these associations. The values represent the square root of the AVE of the latent variables and are the highest in any column or row. The discriminant validity was found to be satisfactory according to the Fornell–Larcker criterion [67].

#### 4.5.4. Indicator Cross-Loading

The cross-loadings of measurement items can also be used to estimate the discriminant validity of the measurement model. As shown in Table 4, each indicator had the highest factor loading on the corresponding construct, suggesting that the measurement model is valid and reliable for structural path modeling.

#### 4.5.5. Discriminant Validity (HTMT)

Discriminant validity assesses the measurement by anticipating the number of uncorrelated constructs [68]. The cross-loadings and the Fornell-Larcker criterion are traditionally used to assess the discriminant validity of indicators [64]. However, [69] recently questioned the reliability of these approaches for having low sensitivity in detecting discriminant validity problems and advocated an alternative method of assessing correlations using the heterotrait–monotrait ratio (HTMT). In Table 5, none of the inter-construct correlations are greater than 0.85, indicating that the discriminant validity was acceptable in this study.

#### 4.5.6. Assessing the Structural Model

The structural model shows the relationship between the evaluated constructs. The inner variance inflation factor (VIF) values were used to assess multicollinearity in the structural equation model. There was no multicollinearity, as all VIF values were less than five.

R^2^ is a measure of the variance in endogenous variables and a measure of the prediction accuracy of the model [70]. Based on Figure 3, the R^2^ value for market stability and financial aid is 0.128, implying that project-related and material-related impacts account for 12.8% of the variance in market stability and financial aid. The R^2^ value for supply chain and project support is 0.188, implying that project-related and material-related impacts account for 18.8% of the variance in supply chain and project support. The R^2^ score for information and legislation is 0.047, implying that project-related and material-related impacts account for 4.7% of the variance in information and legislation.

Bootstrapping was conducted to assess the significance of the relationships between the constructs. At the 5% and 1% levels of significance (for a two-tailed test), the t-statistic values must be equal to or greater than the cutoff values of 1.96 and 2.58, respectively [60]. The results showed that the path coefficients for H1 and H5 were significant at the 5% and 1% levels, implying that these hypotheses are supported (Table 6). These results suggested that project-related impacts have a positive correlation with market stability and financial aid with a path coefficient of 0.272. It is evident that the material-related impacts influence the need for supply chain and project support with a positive path coefficient of 4.069. However, H2, H3, H4, and H6 had low path coefficients with t-values less than 1.96, indicating that they are not supported.

The effect size of R^2^ was used to assess the strength of the variance. the effect size (f^2^) indicates how much one independent construct contributes to explaining a certain dependent construct in terms of R^2^. The construct effect size is small if 0.02 ≤ f ^2^ < 0.15, medium if 0.15 ≤ f ^2^ < 0.35, and large if f ^2^ ≥ 0.35 [71]. Table 6 shows the effect size estimates for some of the constructs that could be estimated. Accordingly, project-related impacts have a small effect size on market stability and financial aid (0.032) and information and legislation (0.022); material-related impacts have a medium effect size (0.192) on supply chain and project support.

### 4.6. Validation by Industry Experts

To validate the study’s findings, post interviews were performed with selected experts from a wide range of the Malaysian construction industry. In total, seven Malaysian experts from the construction industry were targeted. To ensure that feedback was offered from the perspectives of the main construction project stakeholders, the experts were chosen to include contractors, consultants, and project owners. To ensure the reliability of the interview results, the industry experts must hold senior or managerial positions. Interviewees were carefully chosen to ensure that they were experienced experts in their industries, i.e., with more than 10 years of experience in the construction industry. All the interviews were conducted over the phone.

The industry experts were informed of the study background and the validation process. All experts confirmed that the study findings were reasonable. In addition, the experts believed that the study findings could provide insights to policymakers and researchers. The study findings can support advocates, organizations, and policymakers in making suitable management decisions to reduce the pandemic’s impacts on the construction industry. Furthermore, the experts were asked to provide possible explanations for the results to gain in-depth understandings of the findings described in the following subsections.

## 5. Discussions

In examining the impacts of COVID-19 on the construction industry, this study provides significant evidence to support hypotheses H1 and H5. Accordingly, project-related impacts have a significant relationship with market stability and financial aid, material-related impacts have a significant relationship with supply chain and project support, and project-related impacts have a small effect size on information and legislation.

### 5.1. Relationship between Project-Related Impacts and Market Stability and Financial Aid (H1)

Table 6 demonstrates that project-related impacts have a significant relationship with market stability and financial aid. From the analysis, one of the indicators of market stability and financial aid is providing financial aid, including funding, grants, and tax relief. Construction organizations can use financial aid to reduce the pandemic’s impacts on productivity by providing adequate PPE and paying workers. In addition, financial aid can save organizations from bankruptcy, even when most construction projects are delayed, postponed, or canceled [16,72]. The post-survey interviews echoed this sentiment. Policymakers prioritize the construction of new hospitals and clinics over other planned projects in response to the pandemic; new hospitals have been planned specifically for COVID-19. As a result, less critical public construction projects have been postponed. Reduced demand for construction projects can lead to the bankruptcy of construction organizations. Construction organizations in Sweden reported the most bankruptcies during the pandemic [73]. Therefore, providing financial aid to construction organizations is critical to avoid closure and loss of jobs.

Another indicator of market stability and financial aid is mandating aid for construction loans, including deferring loan payments, reducing interest rates, and maintaining liquidity access and credit provisions. Providing liquidity such as loans or another form of credit or deferring loan payments is one of the ways to help businesses survive and retain workers [74]. Financial institutions can lift reserve requirements, allowing construction organizations to increase their loan capacities. When loans are easily approved, private projects and the demand for construction-related work increase. Apart from financial support, policymakers can mandate aid to sustain the economy and maintain employment. The post-survey interviewees emphasized the role of financial institutions in the construction industry.

### 5.2. Relationship between Material-Related Impacts and Supply Chain and Project Support (H5)

From the analysis (Table 6), material-related impacts have a significant relationship with supply chain and project support. The COVID-19 pandemic disrupted the construction material supply chain, which consists of multiple layers of businesses. The construction material supply chain is vulnerable, especially for construction organizations that rely on a single source in a country or geographical region. Suppliers have been notifying contractors and subcontractors of delivery delays or cancellations for some construction materials as a result of the pandemic [21,75]. Non-operation of manufacturing plants and logistics to contain the outbreak have resulted in the unavailability of some construction materials for export. Shutdowns of local manufacturing plants and trucking companies have affected the construction material supply chain. As a result, project delays and schedule disruptions have resulted from material shortages.

The indicators in the supply chain and project support construct include diversification and restructuring of the existing supply chain. Some organizations may find local sources and suppliers to minimize future interruptions from border closures. Creating a flexible supply chain that can adapt quickly to engage alternative suppliers will help construction organizations address similar impacts. Approaches such as restructuring and diversifying the supply chain can help construction organizations operate normally during a pandemic. Policymakers should identify response strategies that reduce construction material prices in the local market during the pandemic to help the construction industry.

### 5.3. Effect Size of Project-Related Impacts on Information and Legislation (H3)

Table 6 indicates H3 is not supported; however, project-related impacts have a small effect size on information and legal support. The indicators included in information and legal support constructs are creating a website with COVID-19 policies and response mechanisms and mandating COVID-19 as a force majeure, indicating that the impacts of COVID-19 on the construction industry must be addressed through response strategies other than financial aid. The studies conducted in [76,77] proposed that AEC organizations update their operations to ensure that construction projects comply with recent regulations and guidelines. Thus, construction organizations must have consistent and reliable sources for the latest policies and procedures to avoid confusion in construction project operations. Policymakers can develop websites outlining current policies and guidelines for construction organization reference.

As the COVID-19 pandemic is unpredictable and unprecedented, policymakers should consider mandating the pandemic as a force majeure to help construction organizations, which the post-survey interviewees concurred with. The term force majeure refers to unpredictable events beyond reasonable control that hinder the fulfillment of the terms of a contract. In addition to lockdown durations, policymakers should integrate any reduction in labor productivity in force majeure clauses, this integration is necessary as construction organizations face reduced labor productivity with newly established standard operating procedures (SOPs) in the pandemic [20,78]. SOPs include reduced workers on construction sites due to social distancing and working restrictions for symptomatic workers [16,78]. Construction organizations still face challenges if a force majeure is established based on lockdown durations. For example, if a government shuts down construction for three months and allows construction projects to resume at 50% productivity, construction organizations require six months to catch up. Policymakers should establish force majeure clauses based on allowable productivity and lockdown durations.

## 6. Conclusions

Although previous studies have investigated the impacts of COVID-19 on the construction industry and global, as well as local, response strategies, there is little empirical work that evaluates the relationships between COVID-19 impacts and response strategies. To fill that gap, an empirical survey of 107 AEC professionals in the Malaysian construction industry was conducted in this study. The collected data were analyzed using the Kruskal–Wallis test and EFA. Furthermore, PLS–SEM was used to examine the relationships between the impacts and response strategies. The study findings revealed significant relationships between the impact and response strategies’ constructs. A significant relationship was found between project-related impacts and market stability and financial aid. A significant relationship was also found between material-related impacts and supply chain and project support.

The model and findings in this study can be of great value and utility for researchers, policymakers, and advocates seeking empirical quantitative evidence and explanations of the COVID-19 impacts in need of response strategies in the construction industry. A clear understanding of the impacts in need of response strategies is beneficial in successfully addressing the pandemic. Awareness of impacts that are significantly correlated with response strategies can aid policymakers and advocates in devising response strategies that reduce the impacts to the construction industry. The key contribution of this study is the development of a quantitative model that explicates how different impacts influence the need for a response strategy in the construction industry.

### 6.1. Theoretical Implications and Contributions

The findings of this study have several theoretical implications. This study contributes to the body of knowledge on construction engineering and management by identifying the impacts of COVID-19 on the construction industry and response strategies to address these impacts. A clear understanding of the impacts can help to develop appropriate response strategies. This study echoes the suggestion in previous works that specific response strategies should be developed to address the impacts of COVID-19 on the construction industry [21]. This study also advances knowledge accumulation concerning appropriate response strategies to address the impacts of COVID-19 on the construction industry by empirically modeling the relationship between pandemic impacts and response strategies.

To the best of our knowledge, no study has modeled the relationships between the impacts of COVID-19 on the construction industry and the response strategies. Researchers can use the methodology derived in this study to model the relationships between impacts and response strategies in other countries and/or industries. Finally, this study develops a deeper understanding of the impacts in need of response strategies, empirically confirms that COVID-19 has affected the construction industry and supports previous studies that suggest that COVID-19 has negatively affected the construction industry through project delays, labor shortages, and increased material prices [13,21,76].

### 6.2. Managerial Implications

In addition to the theoretical implications, this study’s findings have several managerial implications that can help in addressing the impacts of COVID-19 and mitigate similar impacts in the future. The following recommendations are made for policymakers:

- Provide or mandate financial aid, including deferring loan payments to sustain the economy and enable continued employment. Financial aid can help AEC construction organizations use available resources for other expenses, including employee salaries, equipment, and machinery. Financial aid can include reducing additional project expenses resulting from the pandemic, such as renewal fees for hiring foreign workers, as pointed out by the post-survey interviewees.
- Develop plans to avoid future supply chain disruption. For example, a new policy can be established to reduce construction material prices in the local market during a pandemic to help AEC organizations operate as normally as possible. Post-survey interviewees reported that construction material prices had increased several times during the pandemic, usually due to the increased demand for construction materials when construction projects are allowed to resume after lockdowns.
- Develop a force majeure clause based on construction productivity instead of time with the help of an economist. Several post-survey interviewees reported that approvals for force majeure, extension of time (EOT), and payments should be efficient even for remote applications (from home).
- Create a website providing current policies and guidelines and up-to-date and reliable information to industry stakeholders. The post-survey interviewees suggested that this is critically important. Policymakers should centralize all information under one government agency.

Furthermore, the top management of construction organizations can develop action plans to mitigate the impacts and reduce the severity of similar impacts. Construction organizations can diversify suppliers, avoid having too many suppliers concentrated in one area, and find local vendors to minimize future interruptions due to border closures. Construction organizations can also lock in supplier bids for longer periods to protect against unforeseen price inflation and negotiate favorable terms with suppliers and subcontractors [79].

### 6.3. Limitations and Future Research

With the relevance of the findings, this study also has some limitations to be explored in future research. First, the sample size was small (N = 107). Moreover, PLS–SEM and bootstrapping techniques reduced the potential problems caused by the small sample size. Future research can repeat this study with a larger sample size to validate the model. Second, the data may illustrate the context of a single nation, i.e., Malaysia. Thus, applications of the findings in other countries should be considered with caution, and appropriate adjustments should be made. A wider scope of data collection across different countries and regions would enhance the representation of the pandemic’s impacts and response strategies. Future research could be conducted in different countries to compare the similarities and differences of the model. Third, the nonprobability sampling approach was used because there was no sampling frame for this study. Notwithstanding the inherent limitations, this sampling approach can be used to obtain a representative sample [80]. The approach has been recognized as appropriate when respondents are not randomly selected from an entire population but are selected based on their willingness to participate in the study [81]. Although these limitations are observed, the study findings provide new and valuable insights into the relationships between COVID-19 impacts and response strategies.

## Figures and Tables

**Table 1:** Results of EFA for COVID-19 impacts and response.

Constructs	Code	Indicators	Factor Loadings	Variance Explained	Cronbach Alpha
COVID-19 impacts					
Project-related impacts	IMP9	Reduced number of private projects	0.723	22.243	0.844
	IMP3	Reduced number of public projects	0.630		
	IMP12	Reduced foreign investment in the construction industry	0.556		
	IMP11	Reduced demand on construction-related works	0.554		
	IMP4	Downsizing of existing projects	0.553		
	IMP10	Reduced construction productivity	0.552		
	IMP5	Reduced morale among project team members	0.524		
Material-related impacts	IMP8	Shortage of materials	0.804	9.401	0.810
	IMP6	Disruption in the supply chain	0.779		
COVID-19 response strategies					
Market stability and financial aid	RES10	Provide more financial aids	0.757	42.61	0.854
	RES14	Provide incentives to motivate individuals working at construction sites	0.691		
	RES19	Mandate aids for construction loans	0.655		
	RES21	Provide infrastructure investment budgets to local governments	0.648		
	RES13	Ensure payments for public projects are on time	0.598		
	RES18	Speed up the approval processes for construction work resumptions	0.554		
Supply chain and project support	RES12	Restructure existing supply chain	0.710	11.254	0.856
	RES16	Provide help in digitalizing existing construction projects	0.694		
	RES20	Implement the concept of a sharing economy	0.680		
	RES22	Benchmark COVID-19 policies and measures from other countries	0.607		
	RES7	Diversify existing supply chain	0.569		
	RES15	Provide hands-on assistance in implementing SOPs at project sites	0.520		
Information and legislation	RES5	Create a website on COVID-19 policies and response mechanisms	0.811	9.145	0.646
	RES4	Mandate COVID-19 as force majeure	0.578		

**Table 2:** Measurement model assessment.

Constructs	Indicators	Loadings	AVE	CR	CA
Market stability and financial aid	RES10	0.695	0.727	0.838	0.717
	RES19	0.985			
Supply chain and project support	RES12	0.766	0.754	0.858	0.713
	RES7	0.960			
Information and legislation	RES4	0.942	0.723	0.838	0.650
	RES5	0.748			
Project-related impacts	IMP10	0.758	0.554	0.861	0.803
	IMP11	0.716			
	IMP12	0.772			
	IMP5	0.773			
	IMP9	0.701			
Material-related impacts	IMP6	0.925	0.840	0.913	0.810
	IMP8	0.908			

Note: AVE = Average variance extracted; CR = Composite reliability; CA = Cronbach’s alpha; Items removed: items below 0.7: IMP3, IMP4, RES13, RES14, RES18, RES21, RES15, RES16, RES20, RES22.

**Table 3:** Discriminant validity (Fornell-Larcker criterion).

Constructs	Market Stability and Financial Aid	Supply Chain and Project Support	Information and Legislation	Project-Related Impacts	Material-Related Impacts
Market stability and financial aid	0.852	-	-	-	-
Supply chain and project support	0.352	0.868	-	-	-
Information and legislation	0.243	0.241	0.850	-	-
Project-related impacts	0.343	0.172	0.211	0.745	-
Material-related impacts	0.280	0.425	0.164	0.571	0.917

**Table 4:** Cross-loading of the indicators.

Indicators	Market Stability and Financial Aid	Supply Chain and Project Support	Information and Legislation	Project-Related Impacts	Material-Related Impacts
RES10	0.695	0.331	0.343	0.121	0.010
RES19	0.985	0.326	0.197	0.367	0.321
RES12	0.440	0.766	0.160	0.063	0.198
RES7	0.264	0.960	0.242	0.194	0.464
RES4	0.264	0.180	0.942	0.226	0.160
RES5	0.113	0.273	0.748	0.103	0.112
IMP10	0.165	0.213	0.179	0.758	0.525
IMP11	0.210	0.176	0.018	0.716	0.529
IMP12	0.320	0.078	0.242	0.772	0.321
IMP5	0.355	0.097	0.160	0.773	0.444
IMP9	0.151	0.112	0.131	0.701	0.354
IMP6	0.287	0.381	0.195	0.590	0.925
IMP8	0.224	0.400	0.101	0.450	0.908

**Table 5:** Discriminant validity (HTMT).

Constructs	Market Stability and Financial Aid	Supply Chain and Project Support	Information and Legislation	Project-Related Impacts	Material-Related Impacts
Market stability and financial aid	-	-	-	-	-
Supply chain and project support	0.633	-	-	-	-
Information and legislation	0.412	0.371	-	-	-
Project-related impacts	0.340	0.205	0.266	-	-
Material-related impacts	0.259	0.494	0.215	0.716	-

**Table 6:** Structural model assessment.

Hypotheses	Relationship	Path Coefficient	*t*-Value	Decision	f^2^	Effect
H1	Project-related impacts → Market stability and financial aid	0.272	2.382 *	Supported	0.032	Small
H2	Project-related impacts → Supply chain and project support	−0.105	0.778	Not supported	0.007	No effect
H3	Project-related impacts → Information and legislation	0.174	0.937	Not supported	0.022	Small
H4	Material-related impacts → Market stability and financial aid	0.125	0.885	Not supported	0.018	No effect
H5	Material-related impacts → Supply chain and project support	0.485	4.069 **	Supported	0.192	Medium
H6	Material-related impacts → Information and legislation	0.065	0.407	Not supported	0.001	No effect

Note: f^2^ = effect size; * *p* < 0.05; ** *p* < 0.01.

## Data Availability

The data presented in this study are available on request from the corresponding author. The data are not publicly available due to some data being proprietary or confidential in nature. Therefore, the data may only be provided with restrictions (e.g., anonymized data).

## Appendix A

**Table A1:** List of impacts of COVID-19 identified from the literature and interviews.

Code	Pandemic Impact	Source	Interview
IMP1	Higher rejection rate of project financing	[82,83,84,85,86]	
IMP2	Shortage of labor	[87,88,89,90,91,92,93,94,95,96,97]	**✓**
IMP3	Reduced number of public projects	[93,98,99,100,101,102]	
IMP4	Downsizing of existing projects	[87,103,104,105,106]	**✓**
IMP5	Reduced morale among project team members	[107,108,109,110,111]	
IMP6	Disruption in the supply chain	[93,100,102,112,113]	**✓**
IMP7	Termination of existing projects	[22,114]	**✓**
IMP8	Shortage of materials	[88,93,115]	
IMP9	Reduced number of private projects	[93,98,99,100,101,102]	
IMP10	Reduced construction productivity	[90,116,117,118,119]	**✓**
IMP11	Reduced demand on construction-related works	[95,96,101,120,121]	
IMP12	Reduced foreign investment in the construction industry	[120,122,123,124]	

**Table A2:** List of response strategies of COVID-19 identified from the literature and interviews.

Code	Pandemic Response Strategies	Source	Interview
RES1	Allow construction projects to work around the clock (24/7)	[100,102,125,126,127,128,129,130,131,132]	**✓**
RES2	Facilitate the movement of workers from other sectors to the construction sector (e.g., provide free training related to the construction industry)	[133]	
RES3	Have regular townhall sessions on COVID-19 policies and response mechanisms		**✓**
RES4	Mandate COVID-19 as force majeure	[134]	**✓**
RES5	Create a website on COVID-19 policies and response mechanisms	[115,125,126,128,129,135,136,137]	**✓**
RES6	Form a special task force to provide support in maneuvering COVID-19 (ex. in terms of SOP guidelines, alternative procurement methods)	[100,115,129,132,137,138]	
RES7	Diversify existing supply chain	[102,115,125,126,127,130,139,140]	
RES8	Develop platforms to facilitate the generation of alternative revenues	[100,138,141]	
RES9	Initiate Corporate Social Responsibility (CSR) programs targeting COVID-19	[115,138,142]	
RES10	Provide more financial aids (e.g., funding, grants, tax relief)	[115,131,138,139,141,143,144,145]	**✓**
RES11	Facilitate the promotion of local construction materials		**✓**
RES12	Restructure existing supply chain	[115,131,146]	
RES13	Ensure payments for public projects are on time	[115,131,138,139,143,144,145]	
RES14	Provide incentives to motivate individuals working at construction sites	[100,125,126,127,128,129,130,131,139,140,147]	**✓**
RES15	Provide hands-on assistance in implementing SOPs at project sites	[138,148,149]	**✓**
RES16	Provide help in digitalizing existing construction projects	[100,126,135,136,138,141,150]	
RES17	Develop employee assistance programs that fit all types of working groups	[133,145,151]	
RES18	Speed up the approval processes for construction work resumptions	[115]	
RES19	Mandate aids for construction loans (ex. defer loan payments, reduce interest rates, maintain liquidity access/credit provisions)	[115,131,138,139,141,143,144,145,152]	**✓**
RES20	Implement the concept of a sharing economy	[125]	**✓**
RES21	Provide infrastructure investment budgets to local governments	[115]	**✓**
RES22	Benchmark COVID-19 policies and measures from other countries	[100,102,115,126,129,135,137,138,147,150,152]	

## Appendix B. The Questionnaire Survey Used in This Study

COVID-19 impacts and response strategies on Malaysian construction industry.

### Appendix B.1. Respondent’s Profile

Instruction: Please provide the following information.

Your type of organization:

1. Client (e.g., government, developers)
2. Consultant
3. Contractor
4. Others: __________

Years of experience in the construction industry:

1. Less than 2 years
2. 2–5 years
3. 6–9 years
4. More than 10 years

Most of your recent projects are:

5. Building construction (residential)
6. Building construction (non-residential)
7. Industrial construction
8. Infrastructure construction

### Appendix B.2. Impacts and Response of COVID-19 on Malaysia’s Construction Industry

**Table A3:** Please rate the criticality of the following COVID-19 impacts on Malaysia’s construction industry.

COVID-19 Impacts	Criticality				
The COVID-19 impacts were listed in random order using one of the online survey platform features	Not Critical	Slightly Critical	Moderately Critical	Critical	Not Critical
	Not Critical	Slightly Critical	Moderately Critical	Critical	Not Critical
	Not Critical	Slightly Critical	Moderately Critical	Critical	Not Critical
	Not Critical	Slightly Critical	Moderately Critical	Critical	Not Critical
	Not Critical	Slightly Critical	Moderately Critical	Critical	Not Critical

**Table A4:** Please indicate and rate any additional COVID-19 impacts on Malaysia’s construction industry.

Additional COVID-19 Impacts	Criticality				
Additional COVID-19 impacts to be added by survey respondents	Not Critical	Slightly Critical	Moderately Critical	Critical	Not Critical
	Not Critical	Slightly Critical	Moderately Critical	Critical	Not Critical
	Not Critical	Slightly Critical	Moderately Critical	Critical	Not Critical

**Table A5:** Please rate the criticality of the following response strategy on Malaysia’s construction industry.

Response Strategies	Criticality				
The response strategies were listed in random order using one of the online survey platform features	Not Critical	Slightly Critical	Moderately Critical	Critical	Not Critical
	Not Critical	Slightly Critical	Moderately Critical	Critical	Not Critical
	Not Critical	Slightly Critical	Moderately Critical	Critical	Not Critical
	Not Critical	Slightly Critical	Moderately Critical	Critical	Not Critical
	Not Critical	Slightly Critical	Moderately Critical	Critical	Not Critical

**Table A6:** Please indicate and rate any additional response strategy.

Additional Response Strategies	Criticality				
Additional response strategies to be added by survey respondents	Not Critical	Slightly Critical	Moderately Critical	Critical	Not Critical
	Not Critical	Slightly Critical	Moderately Critical	Critical	Not Critical
	Not Critical	Slightly Critical	Moderately Critical	Critical	Not Critical
